# The Role of Macrophages in Acute and Chronic Wound Healing and Interventions to Promote Pro-wound Healing Phenotypes

**DOI:** 10.3389/fphys.2018.00419

**Published:** 2018-05-01

**Authors:** Paulina Krzyszczyk, Rene Schloss, Andre Palmer, François Berthiaume

**Affiliations:** ^1^Biomedical Engineering, Rutgers University, The State University of New Jersey, Piscataway, NJ, United States; ^2^Chemical & Biomolecular Engineering, The Ohio State University, Columbus, OH, United States

**Keywords:** macrophages, chronic wounds, wound healing, inflammation, skin regeneration

## Abstract

Macrophages play key roles in all phases of adult wound healing, which are inflammation, proliferation, and remodeling. As wounds heal, the local macrophage population transitions from predominantly pro-inflammatory (M1-like phenotypes) to anti-inflammatory (M2-like phenotypes). Non-healing chronic wounds, such as pressure, arterial, venous, and diabetic ulcers indefinitely remain in inflammation—the first stage of wound healing. Thus, local macrophages retain pro-inflammatory characteristics. This review discusses the physiology of monocytes and macrophages in acute wound healing and the different phenotypes described in the literature for both *in vitro* and *in vivo* models. We also discuss aberrations that occur in macrophage populations in chronic wounds, and attempts to restore macrophage function by therapeutic approaches. These include endogenous M1 attenuation, exogenous M2 supplementation and endogenous macrophage modulation/M2 promotion via mesenchymal stem cells, growth factors, biomaterials, heme oxygenase-1 (HO-1) expression, and oxygen therapy. We recognize the challenges and controversies that exist in this field, such as standardization of macrophage phenotype nomenclature, definition of their distinct roles and understanding which phenotype is optimal in order to promote healing in chronic wounds.

## Clinical and economic significance of chronic wounds

Following surgical incisions and minor lacerations, diabetic, venous, and pressure ulcers are the most common wounds on a global scale (MedMarket Diligence, 2012, 2015). Whereas, a majority of surgical incisions and lacerations are categorized as acute wounds and often heal with minimal complications, ulcers are chronic wounds that resist healing and require expensive treatments. Furthermore, as surgical wounds become less of a problem due to the advances of minimally invasive surgery, chronic wounds are on the rise, as they often occur in growing populations, such as the elderly, obese, and diabetic. In recent years, there were ~4.5, 9.7, and 10 million pressure, venous, and diabetic ulcer wound patients globally (MedMarket Diligence, 2012, 2015). The numbers of pressure and venous ulcers are rising at rates of 6–7% annually, and growth is even larger for diabetic ulcers (9%) due to the increased incidence of diabetes in the developed world. Unfortunately, the staggering number of chronic, non-healing wounds is growing much faster than the emergence of new, effective therapies.

Standard wound care involves patient and wound assessments, offloading, debridement of necrotic and infected tissue, treatment with antibiotics, and regular wound dressing changes (Falanga, 2005; Frykberg and Banks, 2015). Advanced therapies are available for wounds that do not improve after several weeks of standard care. These include negative pressure wound therapy, topically applied platelet-derived growth factor (PDGF) (Regranex), acellular extracellular matrices (Integra, Matristem, Theraskin), and bioengineered cell-containing therapies (Apligraf, Dermagraft), to name a few. Other possible treatments include hyperbaric or topical oxygen treatment in order to restore oxygen to the wound. In the case of wounds in which infection and severe tissue damage cannot be controlled, the effects of which may otherwise be life-threatening, amputation is performed. In fact, two thirds of all lower-limb amputations are due to diabetic ulcers (Sen et al., 2009). Since many chronic wounds do not improve with standard care, treatment quickly becomes expensive with the introduction of advanced therapies, and sometimes amputation.

With so many people suffering from chronic wounds, and so many failed attempts at treating them, it is not surprising that wound care costs are also enormous. In the United States, over $25 billion dollars are spent annually on chronic wound care (Sen et al., 2009). In England, costs for pressure ulcer treatment can reach up to 6500 pounds per patient (>$8,000 U.S. dollars) (Posnett and Franks, 2008). Similarly, in the United States, the average cost of Medicare spending on pressure and arterial ulcers in 2014 was $3696 and $9015 per patient, respectively—the two most expensive of all types of wounds included in the study (Nussbaum et al., 2018). Furthermore, each amputation procedure can cost well over $35,000 (Gordois et al., 2003; Sen et al., 2009; Carls et al., 2011). Due to the increasing prevalence of diabetes in the U.S., the total cost of diabetic ulcer care has also drastically increased in the past 20 years (Nussbaum et al., 2018). There is an urgent need to understand the pathophysiology of non-healing wounds in order to develop effective therapies that restore their ability to resolve and heal.

## The wound healing process and chronic vs. acute wounds

Chronic wounds fail to heal, despite the use of current therapies, because they are stalled in the early inflammatory state within the wound healing stages (Zhao et al., 2016). In contrast, acute wounds progress through this process in a timely manner as they heal.

The wound healing process is composed of three overlapping phases: inflammation, proliferation and remodeling (Singer and Clark, 1999; Baum and Arpey, 2005; Liu and Velazquez, 2008). After skin injury occurs, platelets are activated at the site of blood vessel rupture and promote clot formation to stop blood loss. Platelets also release factors that attract immune cells from the circulation into the wound. This marks the beginning of the inflammatory phase. Polymorphonuclear neutrophils are first to arrive, followed by monocytes that quickly differentiate into macrophages (Sindrilaru and Scharffetter-Kochanek, 2013). Neutrophils produce high levels of reactive oxygen species (ROS), proteases and pro-inflammatory cytokines to sanitize the wound. When this process is complete, neutrophils apoptose and become phagocytosed by the newly arrived macrophages. Macrophages will also phagocytose bacteria and debris in order to clean the wound (Frykberg and Banks, 2015). During this time, the wound is sterilized and prepared for tissue regrowth, which occurs in the proliferative phase (Baum and Arpey, 2005). As the name indicates, wound cells proliferate and migrate during this phase, in order to regenerate the lost tissue. This includes endothelial cells, fibroblasts and keratinocytes. A preliminary, vascularized extracellular matrix (ECM), called the granulation tissue (GT), is laid down and keratinocytes migrate upon it to close the wound. During remodeling, the final phase of wound healing, ECM within the granulation tissue matures and increases in mechanical strength (Falanga, 2005). Wound healing is complete following apoptosis of myofibroblasts and vascular cells, leaving behind a collagen-rich scar (Zhao et al., 2016).

In chronic wounds, the proliferative and remodeling stages do not readily occur (Zhao et al., 2016). The wound remains in the inflammatory phase, which does not favor tissue regeneration, and therefore, the wound cannot heal (Frykberg and Banks, 2015). Targeting and correcting cellular and molecular causes of prolonged inflammation in chronic wounds may be an effective method to return them to healing states.

## The general role of macrophages in wound healing

There is considerable evidence that macrophages are key regulators of the wound healing process, during which they take on distinct roles to ensure proper healing. It is well-established that the phenotype of macrophages evolves with the stages of wound healing (Mosser and Edwards, 2008; Ferrante and Leibovich, 2012). Initially, pro-inflammatory macrophages, traditionally referred to as “M1” macrophages, infiltrate after injury in order to clean the wound of bacteria, foreign debris and dead cells. In acute wounds, as the tissue begins to repair, the overall macrophage population transitions to one that promotes anti-inflammatory effects (traditionally and collectively referred to as “M2” macrophages), and the migration and proliferation of fibroblasts, keratinocytes and endothelial cells to restore the dermis, epidermis and vasculature, respectively. This process will eventually close the wound and produce a scar. Macrophages also play particularly important roles in vascularization, by positioning themselves nearby newly forming blood vessels and aiding in their stabilization and fusion (Fantin et al., 2010; Ogle et al., 2016). In the beginning of the final remodeling phase, macrophages release matrix metalloproteinases (MMPs) to breakdown the provisional extracellular matrix, and then apoptose so that the skin can mature to its original, non-wounded state (Vannella and Wynn, 2017). In chronic wounds, pro-inflammatory macrophages persist without transitioning to anti-inflammatory phenotypes, which is believed to contribute to the impairment in tissue repair (Zhao et al., 2016; Hesketh et al., 2017).

Macrophage phenotype readily changes based on spatiotemporal cues during wound healing, and several different subsets of macrophages, beyond the limited confines of simply *M1* and *M2* (Martinez and Gordon, 2014), have been defined depending on their cell surface markers, cytokine/growth factor/chemokine production, and function (detailed in section Macrophage Phenotypes). The goal of this review is to highlight the importance of macrophages as they pertain to acute and chronic wound healing. The physiology of monocyte recruitment, macrophage differentiation, and their roles in wound healing are also discussed. Evidence toward a stalled pro-inflammatory macrophage phenotype in chronic wounds is also presented. Lastly, examples are provided of several different approaches that have been taken toward attenuating pro-inflammatory (M1-like) macrophages and promoting anti-inflammatory (M2-like) macrophages in order to heal chronic wounds. It is important to note that, due to the complexity of macrophages, there are several unanswered questions and controversial topics within the field. These are discussed throughout the text, and are also summarized in Table 1.

**Table 1:** Guide to discussed macrophage questions/controversies.

**Guide to Discussed Macrophage Questions/Controversies**		
**Topic**	**Questions/Controversy in Literature**	**Related section**
Dermal Macrophages	• What is the contribution of tissue-resident, dermal macrophages to wound healing?	Sections Dermal Macrophages and Skin Appendages, Dermal Macrophages and Wound Healing
Monocyte Recruitment/ Macrophage Differentiation	• Are monocytes pre-programmed to becoming a specific macrophage phenotype prior to entering the wound and accordingly recruited when needed? • Or, does the wound microenvironment dictate monocyte differentiation/macrophage fate?	Section Monocyte Recruitment and Differentiation in Wound Healing; Figure 1
*In Vitro* vs. *In Vivo* Macrophages	• Do the phenotypes that are defined based on *in vitro* studies translate into *in vivo* wound macrophages?	Section Macrophage phenotypes
M1/M2 Macrophages	• Do macrophages possess distinct phenotypes with unique functions or do their characteristics form a spectrum? • Can all macrophages transition from one phenotype to another? • Can wound macrophages proliferate *in situ* or are they replenished by newly-infiltrated monocytes?	Section Macrophage Phenotypes; Figure 2; Table 2
Human vs. Murine Models	• How translatable are results obtained from murine models to human chronic wounds?	Section Human vs. Murine Models
Macrophages and Wound Healing	• Which macrophage phenotypes/characteristics are required, and at what time, to result in effective wound healing?	Sections Macrophage Phenotypes During Acute Wound Healing and Macrophage Dysregulation and Chronic Wounds; Figure 2
Targeting Macrophages to Promote Wound Healing	• Are M2-like macrophages the answer to promoting wound healing in all situations? If so, which specific phenotypes/characteristics? • What is the ideal treatment time for chronic wounds in order to promote desired wound macrophages and wound healing?	Section Experimental Therapies and Wound Macrophages; Table 3

## Origins of skin macrophages

Skin macrophages are derived from two different sources: (1) a resident macrophage population established before birth and (2) circulating monocytes that are recruited to areas of injury and differentiate into macrophages (Malissen et al., 2014; Vannella and Wynn, 2017). The first type consists of a self-renewing pool of cells derived from the embryonic yolk sack. These cells, called dermal macrophages, are permanent residents in healthy adult skin, often found in nearby skin appendages. In contrast, during injury, bone marrow-derived monocytes are recruited to the skin, locally differentiate into macrophages and play key roles in wound healing, as discussed previously (Malissen et al., 2014; Vannella and Wynn, 2017).

### Dermal macrophages and skin appendages

There are several types of well-studied tissue-resident macrophages throughout the body, which play important roles in their respective organs. A few examples are microglia in the brain, Kupffer cells in the liver, and alveolar macrophages in the lungs (Davies et al., 2013). Their general roles include debris clearance (e.g., surfactant in alveolar macrophages and red blood cells in Kupffer cells), initiation of the inflammatory response and the return to homeostasis. Due to these general functions of tissue-resident macrophages, it is not surprising that tissue-resident macrophages in skin (dermal macrophages) contribute to the maintenance and renewal of skin appendages during homeostasis, and wound healing.

Dermal macrophages are located in close proximity to hair follicles, in the surrounding connective tissue sheath and help regulate the hair growth cycle (Eichmuller et al., 1998; Christoph et al., 2000; Castellana et al., 2014). One of the activities of macrophages during hair growth is phagocytosis of collagen, to allow for matrix remodeling (Parakkal, 1969). In a murine model, Castellana et al. (2014) found that apoptosis of skin-resident macrophages activated epithelial hair follicle stem cells, which contribute to hair regeneration (Castellana et al., 2014). Macrophage-specific Wnt-signaling was shown to be central to this process; when it was inhibited, hair follicle growth was delayed. Apoptosis of macrophages occurred immediately prior to hair follicles' transition from telogen to anagen—the hair cycle's resting and growth phases, respectively. Although the study did not use a wound healing model, the results have potential implications in regenerating hair in healing skin. In contrast, Osaka et al. 2007 used a wound model (full-thickness murine wounds) to study signaling pathways and macrophage activation during subsequent hair growth (Osaka et al., 2007). They found that apoptosis signal-regulating kinase 1 (ASK1) is important for efficient hair regrowth; ASK1-deficient mice exhibited delayed hair regeneration following wounding. ASK1 has previously been shown to be increased in the epithelial layer of wound peripheries in rats (Funato et al., 1998). ASK1-deficient mice also had dysregulated macrophage function; less macrophages were recruited to the wound site and several chemotactic and activating factors (IL-1β, TNF-α) were downregulated (Osaka et al., 2007). When bone-marrow derived, cytokine-stimulated macrophages were introduced to the wounds via intracutaneous injection in both ASK1+ and ASK1- mice, hair growth was stimulated.

These studies highlight the importance of dermal macrophages in hair growth, which can have many implications in the development of future therapies that promote wound healing along with hair regeneration. Although there is more research linking macrophages to hair follicles rather than sweat or sebaceous glands, there is still evidence that macrophages can also respond to cues in the microenvironment created by these appendages. For example, the type of lipids produced by sebocytes can impact whether local macrophages take on pro- or anti-inflammatory characteristics, which could potentially impede or promote healing in that area (Lovaszi et al., 2017). Overall, appendage regeneration remains one of the biggest challenges in wound repair (Takeo et al., 2015). Large wounds that are able to heal have a lack of hair and are unable to produce sweat and oil, which leads to cosmetic deficiencies and discomfort to patients. In general, the relationship between macrophages and skin appendages warrants attention, specifically in the context of wound healing.

### Dermal macrophages and wound healing

A proposed role for tissue-resident macrophages during injury is that they serve as early indicators of injury or invading pathogens. Some of these macrophages express CD4 and are located near capillaries (Malissen et al., 2014). They are first-responders to injury by recognizing damage-associated molecular patterns (DAMPs; e.g., free heme, ATP) and releasing hydrogen peroxide, which initiates a powerful pro-inflammatory cascade (Minutti et al., 2017). In the case of infection, tissue-resident macrophages recognize pathogen-associated molecular patterns (PAMPs; e.g., lipopolysachharide, LPS). Responses to DAMPs and PAMPs result in the recruitment of neutrophils to help fight early infection (Malissen et al., 2014; Minutti et al., 2017). Monocyte-derived macrophages are also recruited to the wounded area to further amplify the inflammatory response (Davies et al., 2013). Although tissue-resident macrophages aid in the recruitment of immune cells, they are not the only cells in the wound (e.g., platelets) that produce chemokines and signals that have this effect. In general, dermal macrophages can be identified by several surface markers, such as CD64+, MERTK+, and CCR2-/low. They are also highly phagocytic and have a slow turnover (Malissen et al., 2014). Near the end of wound healing, during resolution, dermal macrophages self-renew, and clear apoptotic cells as the tissue returns to homeostasis (Davies et al., 2013).

In addition to macrophages, there are also dendritic cells in the skin that are derived from monocytes (e.g., Langerhans cells). These cells share many surface markers with macrophages, including MHCII, F4/80, CD14, and IL-10, which can make it difficult to distinguish them from each other (Malissen et al., 2014; Minutti et al., 2017). Some even consider Langerhans cells as a type of tissue-resident macrophage, as they have a similar gene expression profile (Satpathy et al., 2012; Davies et al., 2013; Doebel et al., 2017; Minutti et al., 2017), and interestingly, a correlation between healing diabetic foot ulcers and increased numbers of Langerhans cells has been reported (Stojadinovic et al., 2013; Doebel et al., 2017). The specific role of Langerhans cells in wound healing—particularly chronic—has yet to be defined, however, they do repopulate the epidermis during re-epithelialization in acute wound models (Stojadinovic et al., 2013).

### Monocyte recruitment and differentiation in wound healing

Whereas dermal macrophages initiate the local inflammatory response and have relatively short-term effects, monocyte-derived macrophages are systemically recruited ~24 h post-wounding (in mice) in order to heighten the inflammatory response and protect the tissue from further damage (Italiani and Boraschi, 2014; Minutti et al., 2017). Monocyte-derived macrophages are initially recruited to the wound by signals from damaged tissue via DAMPs or PAMPs (Sindrilaru and Scharffetter-Kochanek, 2013; Ogle et al., 2016; Vishwakarma et al., 2016). For example, lipopolysaccharide (LPS), a component of the outer membrane of Gram-negative bacteria, is a PAMP that macrophages recognize via binding with toll-like receptor 4 (Bianchi and Manfredi, 2009). This signaling pathway activates the transcription factor, NF-κB, which leads to expression of pro-inflammatory genes. Extracellular DNA, RNA, and ATP, released due to cell death, are examples of DAMPs that signal immune cells and attract them to injury sites (Gallucci, 2016). Monocytes can also be recruited to the wound by chemokines and cytokines downstream of DAMPs/PAMPs, such as IL-1, IL-6, TNF-α, and CCL2 (MCP-1), although in mice, CCL3(MIP-1α) and CCL4(MIP-1β) play this role (Evans et al., 2013).

Multiple monocyte types, categorized as pro-inflammatory and anti-inflammatory, are attracted to the wound site (Ogle et al., 2016). The former, sometimes defined as “classical” monocytes, are derived from the bone marrow and spleen, increase in concentration in the bloodstream following injury, and are CD14^+^CD16^−^ (human) or Ly6C^+/high^ (mice) (Ogle et al., 2016; Boyette et al., 2017). Surface cell adhesion molecules, such as the α_4_β_1_ integrin and CD62L, are responsible for recruiting these cells from the circulation to the blood vessel wall. When there is no injury, pro-inflammatory monocytes do not tightly adhere. However, in the vicinity of the wound, the local presence of inflammatory chemokines and cytokines, such as CCL2(MCP-1), TNF-α, and IFN-γ, promotes expression of cell adhesion molecules. This facilitates the firm adhesion of pro-inflammatory monocytes to the endothelium and subsequent translocation into the tissue space. In addition to extravasation, another mechanism of monocyte recruitment to wounds is by entering through micro-hemorrhages in damaged blood vessels (Rodero et al., 2014; Minutti et al., 2017). With a half-life of only 20 h (in mice), the numbers of pro-inflammatory monocytes fluctuate with the supply of new cells recruited from the bone marrow and circulation, but reach a peak ~48 h after injury (Yona et al., 2013; Ogle et al., 2016). The second type of recruited monocytes consists of anti-inflammatory monocytes, which have a longer half-life (>2 days, in mice). Human markers include CD14^low/−^CD16^+^ and for mice, Ly6^−/low^. These cells attach to the blood vessel wall via α_L_β_2_ integrin (LFA-1) and L-selection (CD62L). The expression of CD62L enables anti-inflammatory monocytes to crawl on the endothelium even during homeostasis, so that they are nearby to aid in tissue and vascular repair when needed (Auffray et al., 2007; Ogle et al., 2016; Boyette et al., 2017). This suggests that, in addition to tissue-resident macrophages, “resident” monocytes may exist as well. Interestingly, pro- and anti-inflammatory monocytes in mice are attracted to areas of inflammation via different signals: CCR2 vs. CX3CR1-dependent pathways, respectively (Italiani and Boraschi, 2014; Ogle et al., 2016).

Others have used a different nomenclature to group human monocytes into classical (CD14^++^CD16^−^), intermediate (CD14^dim^CD16^++^) and non-classical (CD14^++^, CD16^+^) phenotypes. The classical phenotype is analogous to the pro-inflammatory phenotype previously described, whereas the non-classical phenotype is analogous to anti-inflammatory monocytes (Boyette et al., 2017). Each subset exhibits a different morphology following tissue culture, with classical being the largest and roundest, and non-classical being the smallest and having poor attachment. They each have distinct secretomes and respond to different stimuli to varying degrees. For example, classical and intermediate monocytes readily respond to bacteria-associated signals, whereas non-classical monocytes are much less responsive (Cros et al., 2010). All monocyte subsets are capable of differentiating into M1 and M2 macrophages *in vitro*, however M1 macrophages derived from classical monocytes are the most phagocytic and hence, the “most M1-like” (Boyette et al., 2017). Interestingly, non-classical monocytes can differentiate into macrophages even in the absence of differentiation media. This may support one of the proposed models that monocytes themselves transition from classical to non-classical, before differentiating into macrophages (Ogle et al., 2016; Boyette et al., 2017). So, in addition to the existence of several monocyte phenotypes, each possesses varying potentials to differentiate into different macrophage phenotypes as shown through these *in vitro* studies. This adds further complexity in understanding monocyte recruitment/macrophage differentiation in *in vivo* wound healing, where this process is also not entirely clear.

**Figure 1:**
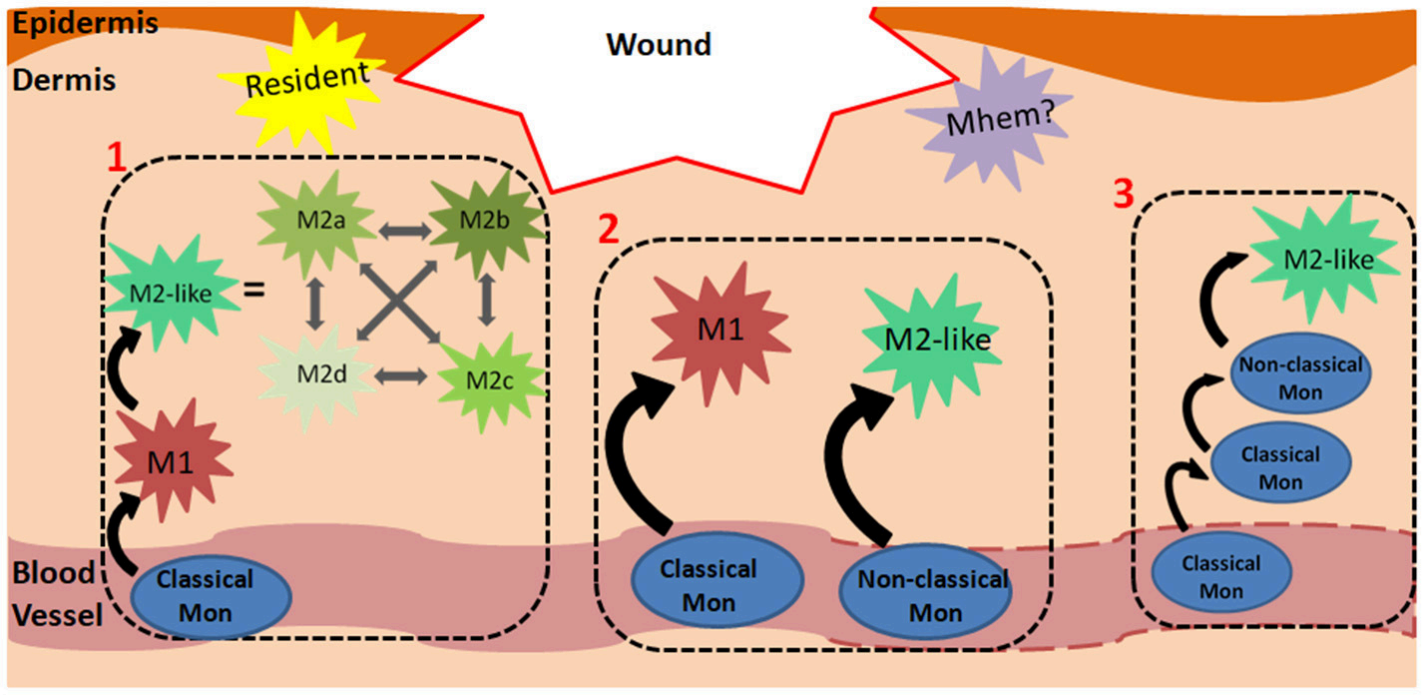
Monocyte-Macrophage Recruitment and Differentiation in Wounds. The mechanism of monocyte recruitment and macrophage differentiation during dermal wound healing can vary depending on spatiotemporal cues. A few models are presented: (1) Classical monocytes in the circulation are primed to differentiate into M1 macrophages following extravasation. In the wound microenvironment, they respond to spatiotemporal cues and can differentiate into any of the M2-like phenotypes, which can transdifferentiate into one another. For brevity, M2a, b, c and d phenotypes are also categorized as M2-like in the remaining processes. (2) Classical monocytes can differentiate into M1 macrophages in the wound. In contrast to the first model, in this panel, macrophages retain the M1 phenotype without further differentiating to M2-like macrophages. Similarly, non-classical monocytes are primed to differentiate into M2-like macrophages and can retain this phenotype. This panel suggests that the final macrophage phenotype is predetermined by the starting monocyte phenotype, and an M1/M2 transition does not occur. (3) This model shows that classical monocytes, rather than macrophages, can also persist in the wound environment for several days, and at a later time, differentiate into non-classical monocytes and then M2-like macrophages. Dashes on the blood vessel indicate that monocytes can exit damaged vasculature via micro-hemorrhages. The yellow star-shape represents resident macrophages, which are established during embryonic development. The purple star-shape represents a possible Mhem phenotype in wounds (analogous to that found in atherosclerotic plaques) which breakdowns hemoglobin and releases anti-inflammatory factors.

In humans, at homeostasis, ~85% of blood monocytes are classical, 5% are intermediate and 10% are non-classical (Italiani and Boraschi, 2014). In inflammatory conditions, classical monocytes differentiate into M1-like macrophages whereas non-classical monocytes aid in tissue repair and differentiate into M2-like macrophages (Figure 1, Process 1). Accordingly, classical monocytes are recruited to wounds to a higher extent following injury compared to non-classical monocytes. There is also evidence that classical monocytes are recruited to the skin for their pro-inflammatory effects, can become non-classical monocytes and eventually differentiate into M2-like macrophages (Crane et al., 2014; Ogle et al., 2016). With several different monocyte and macrophage phenotypes, the recruitment and differentiation processes are complex, especially within dynamic wound microenvironments. It is not surprising that several models of monocyte recruitment and macrophage differentiation during injury have arisen. Although not exhaustive, a few widely discussed models are depicted in Figure 1.

## Macrophage phenotypes

General markers for wound macrophages include CD14^+^, FXIIIA^+^, F4/80 (in mice), CD68 (macrosialin), and lysozyme M (LYZ2). Macrophages can also be identified by their relatively strong autofluorescence, which differentiates them from similar CD14^+^ monocyte-derived dendritic cells (Njoroge et al., 2001; Malissen et al., 2014). In general, primary macrophages have limited proliferative capabilities *in vitro*, although there is evidence that dermal macrophages can self-renew *in vivo* (Davies et al., 2013). In contrast, it is not clear whether monocyte-derived macrophages proliferate *in vivo*, or if they are simply recruited to the site of injury as needed, and apoptose following healing (Murray and Wynn, 2011; Italiani and Boraschi, 2014). Furthermore, their proliferative capability may depend on the particular microenvironment or stage of healing (Italiani and Boraschi, 2014; Murray, 2017).

As described in section The General Role of Macrophages in Wound Healing, two categories of macrophages have been traditionally defined—classically activated, *M1 macrophages* and alternatively activated, *M2 macrophages*. M1 macrophages are typically associated with pro-inflammatory events, whereas M2 macrophages are recognized as anti-inflammatory and pro-regenerative. However, accumulating data suggest that this bipolar M1/M2 definition is grossly oversimplified. It is important to note that M1 and M2 macrophages are not distinct categories, however they form a spectrum in which cells possess varying degrees of M1- or M2-like qualities (Martinez and Gordon, 2014). In support of this view, *in vivo* studies suggest that a heterogeneous population of macrophages exists, with each cell exhibiting a variety of M1 and M2 characteristics (Ogle et al., 2016). Some have even described macrophage activation as a “color wheel,” with classically-activated, wound healing and regulatory macrophages as the primary colors, and the secondary colors representing intermediate macrophage phenotypes (Mosser and Edwards, 2008). As a result, many additional subpopulations of macrophage phenotypes have been described and defined in the literature.

**Figure 2:**
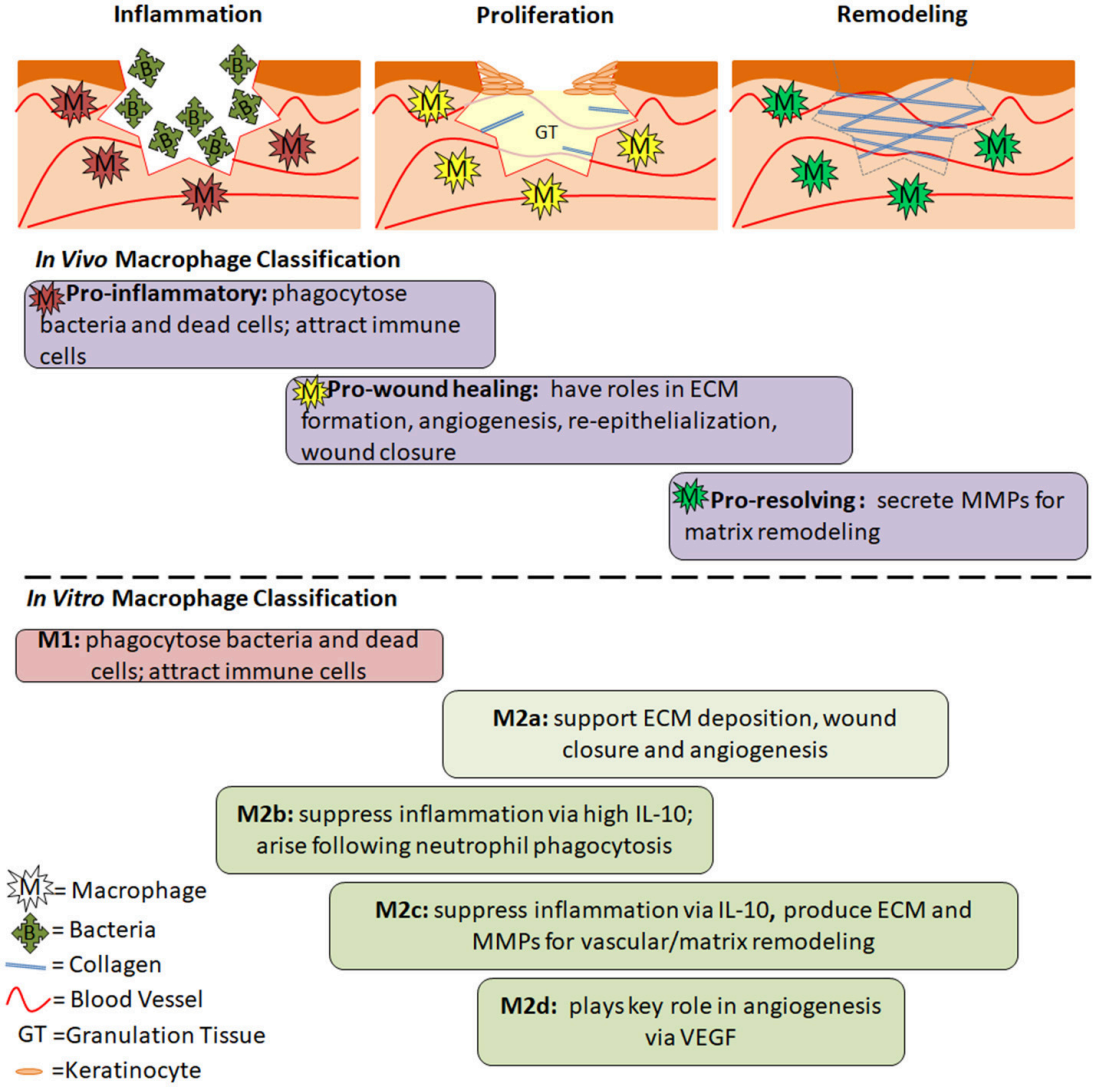
The Role of Macrophage Phenotypes in Wound Healing. Acute wounds progress through the phases of inflammation, proliferation and remodeling as they heal. In **inflammation**, pro-inflammatory macrophages are present. Their role is to phagocytose dead cells and bacteria and prepare the wound for healing. In **proliferation**, pro-wound healing macrophages are present. They secrete factors that aid in angiogenesis, formation of granulation tissue, collagen deposition, and reepithelialization. In **remodeling**, pro-resolving macrophages aid in breakdown of the provisional granulation tissue to allow for maturation of collagen and strengthening of the newly regenerated skin. Below the diagrams are the general roles and timing of different macrophage phenotypes during the wound healing process. Differences between *in vivo* and *in vitro* classifications are separated by the dashed line, however similar roles can be seen between many of the phenotypes. The timing is an estimate based on the role of each phenotype, and has not been experimentally confirmed.

Before discussing the specifics of M1- and M2-like macrophages, a different categorization will be presented in section Pro-inflammatory, pro-wound Healing and pro-resolving Macrophages—one that separates macrophage phenotype based on their role within the wound healing process. In regards to this review, this categorization is more relevant and intuitive, although it is not as widely accepted as the M1/M2 spectrum (section M1/M2 Macrophage Spectrum). Discrete M1/M2 phenotypes are useful *in vitro* when the stimulating molecule is known and experimentally introduced to the system, however this nomenclature is less applicable when discussing *in vivo* macrophages in a wound healing context (Novak and Koh, 2013). All of the macrophages associated with wound healing across both *in vitro* and *in vivo* classification systems are presented in Figure 2, along with their respective roles.

### Pro-inflammatory, pro-wound healing, and pro-resolving macrophages

In agreement with the phases of wound healing, pro-inflammatory macrophages are present shortly after a wound is formed, followed by pro-wound healing macrophages that support cellular growth and proliferation, and finally pro-resolving macrophages that drastically down-regulate the immune response and aid in collagen reorganization and maturation (Murray and Wynn, 2011; Vannella and Wynn, 2017). Pro-inflammatory macrophages produce nitric oxide, ROS, IL-1, IL-6, and TNF-α. They also secrete MMP-2 and MMP-9 in order to break down the extracellular matrix and make room for infiltrating inflammatory cells (Murray and Wynn, 2011). Pro-wound healing macrophages produce elevated levels of growth factors such as PDGF, insulin-like growth factor 1 (IGF-1), VEGF and TGF-β1 (Murray and Wynn, 2011; Vannella and Wynn, 2017), which aid in cellular proliferation, granulation tissue formation, and angiogenesis. They also produce tissue inhibitor of metalloproteinases 1 (TIMP1) in order to counteract MMPs and allow for ECM formation (Murray and Wynn, 2011). Pro-resolving macrophages (aka regulatory macrophages) suppress inflammation via upregulation of IL-10. They also express arginase 1, resistin-like molecule-α (RELMα) programmed death ligand 2 (PDL2) and TGF-β1. MMPs (some evidence pointing toward MMP-12 and MMP-13 specifically) are produced to remodel and strengthen the ECM (Hesketh et al., 2017; Vannella and Wynn, 2017). The function of pro-resolving macrophages is to restore homeostasis while minimizing fibrosis via apoptosis of myofibroblasts, suppression of T cell proliferation and a return to balanced MMP/TIMP levels (Murray and Wynn, 2011). Just as wound healing phases overlap, these different macrophages also share some characteristics with one another. This is especially true for pro-wound healing macrophages which fall between the early and late phases of wound healing, and therefore exhibit characteristics similar to both pro-inflammatory and pro-resolving macrophages.

### M1/M2 macrophage spectrum

Analogous to pro-inflammatory macrophages, M1 macrophages dominate during the pro-inflammatory phase of wound healing, and through their highly phagocytic behavior, serve the role of sanitizing the wound and clearing it of dead tissue. M1 macrophages also activate other immune cells during the early phase of the wound healing process. *In vitro*, M1 macrophages are stimulated by intracellular proteins and nucleic acids released from lysed cells (e.g., IFN- γ), and bacterial components, such as LPS and peptidoglycan (Ferrante and Leibovich, 2012). M1 macrophages express CD86, and produce high levels of (ROS) and pro-inflammatory cytokines, such as interleukins 1 and 6 (IL-1, IL-6), TNF- α, and IFN-γ.

Traditionally defined *M2 macrophages* serve a regenerative role. M2 macrophages are stimulated by IL-4 and IL-13 and express high levels of the mannose receptor (CD206), dectin, interleukin 10 (IL-10), and transforming growth factor β (TGF-β). They produce low levels of pro-inflammatory cytokines such as TNF-α, IL-12, and CXCL8(IL-8) (Ferrante and Leibovich, 2012). It has also been found that interferon regulator factors (IRF4/IRF5) are transcriptional regulators that play a role in differential signaling seen between M2 and M1 macrophages, respectively.

M2 macrophages have been divided into different subtypes according to differential expression of surface markers. These subtypes have been traditionally used and identified *in vitro* to study M2-like macrophages with different characteristics. In *in vivo* studies, this nomenclature is not as widely used to identify macrophages, potentially due to the heterogeneous populations present, which are generated from a variety of stimuli within wounds (Novak and Koh, 2013). Table 2 identifies the different names and markers for each macrophage phenotype. The table is not comprehensive, and it is important to note that marker expression for each phenotype can vary from study to study, hence adding to the complexity and difficulty of defining macrophages.

**Table 2:** Macrophage phenotypes and characteristics.

**Phenotype**	**Other nomenclature**	**Nomenclature by activation molecule**	**Markers**	**Other notes**
**M1**	classically activated; pro-inflammatory	–	Surface: CD86, CD68, CD80, MHC-II Secreted: TNF-α, IL-6, IL-12, IL-1β	abundant and persistent in chronic wounds; activated *in vitro* by LPS, peptidoglycans and pro-inflammatory cytokines
**M2**	all M2-phenotypes collectively: alternatively activated; anti-inflammatory	–	–	–
M2a	alternatively activated; wound healing	M(IL-4)	Surface: CD206, arginase (mice), Ym1 (mice) CD163, MHC-II, CD209 Secreted: TGF-β, IL-10, IL-1RA	aid in ECM formation, angiogenesis
M2b	type 2; regulatory	M(Ic)	Surface: CD86, MHC-II Secreted: TNF-α, IL-1, IL-6, IL-10	similar to M1 macrophages, but dampen inflammation
M2c	deactivated; pro-resolving?	M(IL-10), M(GC), M(GC+TGF-β)	Surface: CD86, CD163, CD206 Secreted: IL-10, CD206, TGF-β, MMP-9	involved in vascular and matrix remodeling; some shared characteristics with Mhem
M2d	–	–	Secreted: VEGF, IL-10, IL-12, TNF-α, TGF-β	pro-angiogenic; activated *in vitro* by stimulating adenosine and toll-like receptors
**Mhem**	HA-Mac; Heme-directed macrophage	M(Hb)	Surface: CD163, CD206 Secreted: IL-10 Internal: HMOX-1 gene, activating transcription factor (ATF)	found near hemmorrhaged vessels in atherosclerotic plaques; anti-inflammatory effects
**M4**	CXCL4 derived macrophage	–	Surface: CD206, CD86, Lack CD163 Secreted: IL-6, TNF-α	associated with atherosclerosis in human models; M1-like; low phagocytosis
**Mox**	Oxidized phospholipid derived macrophages	–	Surface: ↓arginase-1 Secreted: ↓MCP-1, ↓TNF-α Internal: HMOX-1 gene, HO-1, sulfiridoxin 1, theoredoxin reductase-1	associated with atherosclerosis in murine models; low phagocytosis; antioxidant properties
**TAMs**	tumor-associated macrophages	–	Surface: CD163, CD206, CD204 Secreted: IL-10, MIF, CXCL12, VEGF, IL-6, IL-23, TGF-β Internal: HIF-1α	located nearby tumors; promote angiogenesis and cell proliferation; M2-like

The previously described M2 macrophages, also known as wound healing macrophages, align with what is now defined as the *M2a subset* (Ogle et al., 2016). M2a macrophages are stimulated by IL-4/IL-13 and exhibit IL-4 receptor α (IL-4Rα) signaling (Ferrante and Leibovich, 2012). CD206 is a distinguishing surface marker and they produce high levels of arginase-1 (in mice), PDGF-BB, IGF-1, and several chemokines (CCL17, CCL18, CCL22; Ogle et al., 2016). M2a macrophages produce collagen precursors and factors that stimulate fibroblasts. Thus, M2a macrophages play a key role in ECM formation, which is required during the proliferative phase of wound healing. They also secrete high levels of PDGF, which is involved in angiogenesis (Spiller et al., 2014).

*M2b* macrophages, which express CD86, CD68, and MHCII, are stimulated by immune complexes and TLF/Il-1 agonists (Ogle et al., 2016; Hesketh et al., 2017). They are also known as type 2 macrophages. M2b macrophages suppress inflammation by increasing IL-10 production, although they also secrete IL-6, IL-β, and TNF, and express high levels of iNOS. M2b macrophages also produce several different MMPs. *In vitro*, macrophages take on an M2b phenotype following phagocytosis of apoptotic neutrophils (Filardy et al., 2010).

*M2c* macrophages are stimulated by glucocorticoids, IL-10 and TGF-β (Roszer, 2015; Garash et al., 2016). They express CD206 and MERTK. M2c macrophages produce high levels of IL-10, MMP-9, IL-1β, and TGF-β, and low levels of IL-12. M2c macrophages also express CD163, which is the hemoglobin receptor. This is important to note, as there exists another macrophage phenotype, called Mhem, that is similarly characterized by high CD163 expression and IL-10 production, albeit stimulated by hemoglobin and typically identified in atherosclerotic plaques (Boyle, 2012). These shared features may indicate that different stimuli can elicit the same, or very similar, macrophage phenotypes that are nevertheless referred to by different names (M2c vs. Mhem). Furthermore, due to their production of ECM and MMPs and hence, their matrix remodeling capability, M2c macrophages may be analogous to the aforementioned pro-resolving macrophages. M2c macrophages are also sometimes referred to as deactivated macrophages as they can arise from M1 macrophages that have “deactivated” the M1 gene profile to become M2c macrophages. M2 macrophages can shift between *a, b* and *c* phenotypes (Ogle et al., 2016).

In contrast to *M2a* macrophages, *M2d* macrophages do not have either elevated mannose receptor (CD206) or dectin-1 expression (Ferrante and Leibovich, 2012). M2d macrophages arise from stimulation by IL-6 and adenosine. They produce high levels of vascular endothelial growth factor (VEGF) as well as IL-10 and TGF-β. They also down-regulate pro-inflammatory TNF-α and IL-12. M2d macrophages are activated by concurrent stimulation of toll-like receptor (via IL-6) and adenosine A_2A_ receptors (Ferrante and Leibovich, 2012; Roszer, 2015).

Several other macrophage types have been defined, however, they tend to be associated with specific diseases, such as atherosclerosis or cancer (Colin et al., 2014; Medbury et al., 2014). For example, Mox, M4 and Mhem arise from macrophages stimulated by oxidated phospholipids, CXCL4 and hemoglobin-haptoglobin complexes, respectively. Although these phenotypes are not typically associated with chronic wounds, it is possible that some wound macrophages have some shared characteristics, especially in regards to Mhem, as hemoglobin-haptoglobin receptor (CD163) expression and cellular regulation of iron are associated with wound healing (Cairo et al., 2011; Sindrilaru et al., 2011; Evans et al., 2013). There also exist cancer-specific macrophages, called tumor-associated macrophages (TAMs), which support tumors by stimulating angiogenesis, aiding in metastasis and inhibiting T-cell anti-tumor responses (Yang and Zhang, 2017). They can differentiate from resident progenitor cells, but are more often derived from recruited monocytes from the blood stream. TAMs are more similar to M2 macrophages, as they produce anti-inflammatory cytokines and promote proliferation and growth to support the tumor microenvironment. These additional, disease-specific macrophages underline the unique plasticity and range of phenotypes and functions that macrophages possess and can exhibit in different microenvironments.

Overall, macrophage nomenclature within this vast spectrum is not yet agreed upon and it is unclear whether the phenotypes are distinct, or even applicable to *in vivo* wound healing (Martinez and Gordon, 2014). It is important to remember that the macrophage population during wound healing is complex; wound macrophages can take on a different phenotype depending on several factors, such as the anatomical location of the wound (foot, lower back), the specific region within the wound (center/edge), the environment (moist, dry) and whether or not the wound is infected (Ferrante and Leibovich, 2012). Unsurprisingly, it is still unclear the exact signals and differentiation cascade required to produce a specific macrophage phenotype (M2a vs. M2b vs. M2c, etc., see Figure 1). Adding further complexity to this question is the fact that these phenotypes exist on a spectrum, and macrophages can easily transition from M1-like to M2-like (and M2a-like, M2b-like etc.). Furthermore, wound macrophage populations are heterogenous, as it is possible for pro- and anti-inflammatory cytokines to be present simultaneously (Novak and Koh, 2013; Martinez and Gordon, 2014). Interestingly, although differentiation of M2a macrophages is stimulated by IL-4 and IL-13 *in vitro*, these cytokines are not present in healing murine wounds that contain M2-like macrophages (Daley et al., 2010), further underlining the disconnect between *in vitro* and *in vivo* models. Although the defined macrophage definitions are useful *in vitro*, they must be regarded with caution when considering macrophage phenotypes in the *in vivo* wound healing process.

### Macrophage standardization efforts

Murray et al. (2014) acknowledge the complexity of the current macrophage nomenclature and provide suggestions for improvement moving forward (Murray et al., 2014). The authors met to attempt to set a foundation toward consolidating and standardizing the wealth of macrophage activation terms and methods that have arisen throughout the years.

Their recommendations include:

1. differentiating murine or human bone marrow/peripheral blood monocytes with CSF-1 or GM-CSF to generate macrophages, and using post-differentiation stimuli IFN-γ and IL-4 to obtain M1 and M2 macrophages, respectively;
2. reporting defined metrics such as tissue culture conditions, media, time, etc., to characterize *in vitro* macrophage cultures;
3. defining the activator for *in vitro* macrophages using the following notation: M(LPS), M(IFN-γ), M (IL-10), etc. and referencing a provided spectrum of M1/M2-like characteristics;
4. the avoidance of the term “regulatory macrophages”, as well as the use of GM-CSF to create M1 macrophages and CSF for M2 macrophages; and
5. use of a combination of markers (cytokines, chemokines, scavenger receptors and more) to describe macrophage state.

The authors not only discussed how to define *in vitro* macrophages, but also macrophages isolated from *in vivo* models. A main point includes encouraging scientists to detail the isolation process in publications. Researchers should also characterize *ex vivo* macrophages and attempt to fit them within the *in vitro* macrophage spectrum defined in the article, in a manner similar to that depicted in Figure 2 in this review. They also acknowledge the differences between interspecies macrophages, and suggest thorough side-by-side comparisons in order to glean information about human macrophage behavior. With more characterization and understanding, scientists will begin to bridge the gap between macrophages from different sources and species.

These guidelines were a vital starting point to tackling the complex challenge of streamlining macrophage nomenclature and research/reporting practices. These standards should be broadly distributed, and scientists should regularly meet to update them. As a result, understanding of macrophage function and behavior will improve across the entire field. This may prime faster advancement in the development of therapies that target macrophages, within chronic wound healing applications and many others.

## Human vs. murine models

Mice are commonly-used animal models for wound healing studies due to their affordability and ease-of-use, however, it is important to acknowledge differences between human and murine skin anatomies, wound healing processes, and immune systems (and hence, macrophage behaviors) (Murray, 2017). In terms of anatomy, mice have more densely-packed hair follicles and thinner epidermal and dermal layers compared to human skin (Pasparakis et al., 2014). It is also generally believed that murine skin heals by contraction—that is, the edges of the wound pull in toward each other, like a drawstring bag, in order to quickly close. In contrast, human skin heals by re-epithelialization, during which keratinocytes crawl over the granulation tissue in order to close the wound. This assumption has recently been revisited, to argue that mice heal both by contraction and re-epithelialization, making them better models for wound healing than previously assumed (Chen et al., 2015).

Diabetic mice are used as *in vivo* chronic wound models, as they exhibit delayed wound healing. Mice are either bred to contain a genetic mutation which results in a diabetes-like phenotype, or it is induced via chemical means, for example, injection with streptozocin (Nunan et al., 2014). Diabetic mouse wounds share several key characteristics in common with chronic wounds in diabetic patients (Blakytny and Jude, 2006). These include decreased nerve count, angiogenesis, granulation tissue formation, and collagen content compared to acute wounds. They both contain higher levels of MMPs and lower levels of TGF-β1, IGF-1, and PDGF. More is actually known about diabetic mouse wounds compared to human diabetic ulcers, due to an increased number of studies and an increased ability to probe and measure tissue characteristics (particularly *ex vivo*). So, whereas there are several studies showing decreased VEGF, FGF, and KGF in diabetic mouse wounds, in human diabetic ulcers, there is both an incomplete panel of measured cytokines and growth factors, as well as less significant trends due to large biological variability.

One discrepancy between murine and human macrophages is their expression of inducible nitric oxide synthase (iNOS) (Mestas and Hughes, 2004). Mouse macrophages readily express iNOS in response to LPS or IFN-γ, and for this reason, it is recognized as an M1 marker in mice. Human macrophages, however, do not over-express iNOS in response to these same stimuli. General markers to identify murine and human macrophages differ as well. In humans, they are CD14 and CD33, and in mice, they are F4/80 and CD11b. Other murine-specific M2 markers include Ym1, FIZZ1, and arginase-1 (Roszer, 2015). Human and mouse macrophages also express different FcR and IgG receptors, which play a bigger role in the immune system as a whole, by linking the adaptive and innate immune systems (Mestas and Hughes, 2004).

The function of specific receptors can also differ between species (Mestas and Hughes, 2004). For example, CD163 is a common M2-like macrophage marker that functions as the hemoglobin-haptoglobin receptor (Etzerodt et al., 2013). In humans, the binding of hemoglobin and haptoglobin significantly increases endocytosis of hemoglobin and activation of downstream signaling pathways. In mice however, haptoglobin does not promote binding of hemoglobin to CD163. Although this may seem insignificant, it is just one specific example of how human and murine macrophages have different mechanisms and behaviors. To overcome these discrepancies, there is a need to conduct thorough, side-by-side experiments (e.g., single-cell and bioinformatics approaches) using monocytes and macrophages from different species and sources (Murray, 2017). Through these efforts, well-informed comparisons can be made across models while taking advantage of their other benefits (affordability, ease-of-use, etc.).

In addition to specific differences between human and murine skin and macrophages, on a whole, it is important to remember that, although diabetic mice are slower to heal than wild type mice, they do eventually heal. Diabetic mice are not an ideal model for non-healing, chronic wound studies, but they do have many fundamental similarities on the tissue and cellular levels, making them a widely-accepted model in current wound healing research (Nunan et al., 2014).

## Macrophage phenotypes during acute wound healing

Except for fetal wounds, which have the capacity to regenerate in the absence of inflammatory response, macrophages are vital for successful adult wound healing (Leibovich and Ross, 1975; Mackool et al., 1998). Studies have shown that the depletion of macrophages in wounded mice results in delayed re-epithelialization, reduced collagen formation and impaired angiogenesis (Goren et al., 2009; Mirza et al., 2009). These effects occurred along with increased levels of TNF-α and decreased VEGF and TGF-β1. Furthermore, in the absence of macrophages, there was a prolonged neutrophil presence and reduced wound contraction (Goren et al., 2009).

Although the general importance of macrophages in wound healing is known, there is still much to learn about the details regarding timing, relative proportion and specific role of each phenotype. Mirza and Koh (2011) isolated macrophages during the wound healing process in mice at Days 5, 10, and 20 post-injury in order to study the temporal phenotype change (Mirza and Koh, 2011). In wild type mice, pro-inflammatory macrophages were detected on Day 5. These macrophages expressed high levels of IL-1β, MMP-9, and nitric oxide synthase (NOS). By Day 10, the expression of these pro-inflammatory factors decreased, concurrent with an increase in expression of anti-inflammatory markers CD206 and CD36 and growth factors IGF-1, TGF-β, and VEGF. Non-diabetic mice had efficient wound repair, achieving wound closure after 20 days, at which elevated expression remained for CD206, CD36, and TGF-β, but not for IGF-1 or VEGF.

Evans et al. (2013) used an acute wound model in humans to better understand the pro- to anti- inflammatory macrophage transition in blisters (Evans et al., 2013). Wounds were chemically induced by application of cantharidin, a topical treatment for warts. Blister fluid was collected 16 and 40 h after injury, to represent the inflammatory and resolving phases of wound healing, respectively. Cell counts from the fluid yielded more monocytes/macrophages at the 40 h time point compared to 16 h. Furthermore, the proportion of CD163+ macrophages increased over 10 fold at the later time point (3.4 vs. 47.6%), indicating that CD163 is strongly associated with the resolution phase of healing. Amounts of inflammatory mediators were also measured in the wound fluid. At the 16 h “inflammatory” time point, there were significantly higher levels of CCL2(MCP-1), CXCL8(IL-8), TNF-α, CCL3(MIP-1α), CCL4(MIP-1β), and CCL11(eotaxin). At the 40 h “resolution” time point, there was significantly more macrophage-derived chemokine (MDC) and TGF-β in the wound fluid. Interestingly, this study reported undetectable levels of IL-10 at either time point, which is surprising, as it is recognized as a cytokine produced at high levels by anti-inflammatory macrophages.

## Macrophage dysregulation and chronic wounds

When macrophages become dysregulated, several wound healing complications can arise, such as the formation of chronic wounds or excessive scarring (Vannella and Wynn, 2017).

Macrophages in chronic wounds have a reduced capability to phagocytose dead neutrophils, which, as a result, accumulate and promote a strong inflammatory environment. Diabetic patients have macrophages with reduced apoptotic clearance activity because of the effects of hyperglycemia and advanced glycation endproducts (Khanna et al., 2010; Hesketh et al., 2017). The act of neutrophil clearance by macrophages can induce the phenotypic switch of M1 macrophages to M2b, and lead to the resolution of inflammation (Filardy et al., 2010; Hesketh et al., 2017). This is one of many reasons as to why chronic wounds may have an abundance of M1 macrophages.

Significantly higher numbers of macrophages are found in the peripheries of venous and diabetic ulcers compared to acute wounds (Loots et al., 1998). In this study, CD68 was used as a general marker to detect macrophages (although other studies define it as an M1 marker, this study did not make that clear). In acute wounds, the number of macrophages was highest at the first time point, and decreased as healing progressed. In contrast, venous and diabetic ulcers had the highest number of macrophages compared to acute wounds. The results of this study also suggested that macrophages are not the only immune cell that is dysregulated in chronic wound healing, as lower numbers of T-cells and higher numbers of B-cells were also observed. Another study also detected high levels of CD68 macrophages in the dermis and wound edges in chronic leg ulcers (Moore et al., 1997). CD16 and CD35 were also measured and defined as “activation-associated markers,” with positive staining denoting the presence of mature macrophages (rather than monocytes) in inflammatory environments. Most of the wounds studied had low expression of these markers (<12%), and the few areas that were positive were near the vasculature, suggesting that other microenvironments in the wound suppress or prevent macrophage activation. Although this study provided information about macrophage presence and marker expression in chronic wounds, particularly in different locations of a single wound, a low patient number (12 patients) was evaluated and results were not compared relative to patients with acute wounds. These early studies provided important histological data on macrophages in human wounds, but did not explicitly discriminate between pro- or anti-inflammatory phenotypes, nor did they measure cytokines or growth factors in the wound environment.

Most *in vivo* studies, especially in humans, do not tend to study macrophages directly via detection of cell markers, but rather indirectly through the cytokines and proteins present in the wound tissue or fluid. Macrophages are major producers of cytokines and growth factors during wound healing, so, based on the identities and amounts measured, a determination can be made on whether the local macrophage population is more pro- or anti-inflammatory (M1/M2-like).

Accordingly, studies show that chronic wound fluid contains high levels of pro-inflammatory cytokines, particularly TNF-α and IL-1β, which were measured to be 100-fold higher compared to acute wounds fluids (mastectomy drain fluids; Tarnuzzer and Schultz, 1996). IL-6 was also elevated, but only 2–4 fold. In contrast, mastectomy fluid had the highest levels IL-1β and IL-6 on Day 1 post-surgery and thereby steadily decreased to Day 7. Interestingly, TNF-α levels remained constant during this time period. This is in agreement with observations by Wallace and Stacey, who also observed higher levels of total TNF-α in chronic wounds (Wallace and Stacey, 1998). Interestingly, the amount of bioactive TNF-α in both healing and non-healing wounds was not significantly different. The amount of bioactive TNF-α did not change as acute wounds closed, suggesting that the regulation of other cytokines may be more important in progressing wound healing. Chronic wound fluid also contains high levels of MMPs, specifically MMP-2 and MMP-9 (Wysocki et al., 1993). MMPs degrade proteins and extracellular matrix and are not favorable for extended periods, as they do not support tissue regrowth in the proliferative phase of wound healing. Macrophages produce MMPs, so they may be responsible for maintaining elevated levels in chronic wounds (Newby, 2008). Specifically, human blood monocytes are stimulated to produce MMP-2 and MMP-9 in the presence of pro-inflammatory signals, such as LPS, IFN-γ, IL-1β, and TNF-α (Zhou et al., 2003; Newby, 2008). Furthermore, the proteases degrade and decrease the bioactivity of growth factors that may be present, hence canceling out their pro-healing benefits as inflammation prevails (Tarnuzzer and Schultz, 1996).

Since there are no non-healing animal models of chronic wounds, diabetic mice are often used as they exhibit delayed wound healing and share several characteristics with human chronic wounds (Blakytny and Jude, 2006; Nunan et al., 2014). Studies investigating wound macrophages show that their function is not properly regulated in diabetic vs. wild type mice, with a prolonged M1 macrophage presence and hence, inefficient transition to the M2 phenotype (Mirza and Koh, 2011). Mirza and Koh found that, although macrophages from non-diabetic mouse wounds had transitioned from a pro- to anti-inflammatory phenotype by Day 10, macrophages from diabetic mice retained pro-inflammatory characteristics. This included two-fold higher levels of pro-inflammatory factors IL-1β and IFN-γ, and ~two-fold lower anti-inflammatory IL-10 in the general wound environment. More specifically, isolated macrophages from Day 10 wounds in diabetic vs. non-diabetic mice had significantly higher mRNA expression of IL-1β and MMP-9 and significantly lower expression of CD206 and CD36. At the same time, they have reduced growth factor production (IGF-1, TGF-β1, VEGF, and IL-10). In non-diabetic wounds, these factors are already present and contributing to key events in wound healing such as cell proliferation and migration, ECM formation, and angiogenesis. Most of the aforementioned cytokine and growth factor trends were retained until Day 20, which is in stark contrast to non-diabetic wounds, which had already healed by this point and long-completed the M1-to-M2 transition. Also interesting to note, is the fact that diabetic wounds contained fewer mature macrophages (more Ly6C expression v. F4/80) even at Day 10, suggesting that the monocyte-to-macrophage transition is impaired and may contribute to delayed wound healing. Overall, diabetic and non-diabetic wound macrophages only started to exhibit significantly different cytokine/growth factor differences by Day 10; at Day 5, they had similar levels. This suggests that between Day 5 and Day 10, non-diabetic mouse wounds are transitioning to the proliferative phase, in accordance with the M1 to M2-like macrophage phenotype change. Diabetic mouse wounds remain highly inflammatory, guided by persistent pro-inflammatory macrophages. Overall, this study provided key evidence of delayed macrophage phenotype transition concurrent with delayed wound healing in diabetic mice.

Other studies have shown prolonged presence of other pro-inflammatory cytokines in diabetic mouse wounds, much longer than seen in wild type mice. One study compared the expression of IL-1β and TNF-α in three different mouse strains: Balb/c, C57BLKS, and db/db (Wetzler et al., 2000). No IL-1β was detected after Day 7 in the first two strains, whereas high levels persisted into Day 13 in diabetic mouse wounds. Similarly, TNF-α was detected at the highest levels in db/db mouse wounds at Day 13, and was completely absent or present at very low levels in the wild type groups at the same time. The diabetic group also retained elevated levels of MIP-2 and CCL2(MCP-1) mRNA and protein into Day 13, whereas both strains of wild type mice had stopped producing those factors by Day 13 or even earlier. MIP-2 and CCL2(MCP-1) are chemoattractants, so their presence continually attracted more macrophages, which was detrimental to healing, as the macrophages that were recruited maintained an M1 phenotype. Again, the prolonged presence of pro-inflammatory/M1 macrophages is a hallmark of delayed wound healing in diabetic mice.

Differential iron regulation by macrophages is another factor that can promote M1/M2 phenotypes (Cairo et al., 2011). M1-like cells store the majority of the iron intracellularly as ferritin, whereas, M2-like macrophages release it to the extracellular environment via the transmembrane channel, ferroportin. Sindrilaru et al. (2011) identified the role of high intracellular iron stores in maintaining M1 macrophages in chronic wounds, particularly chronic venous ulcers (Sindrilaru et al., 2011). The source of iron was hemoglobin from erythrocytes that escape from damaged blood vessels and enter the wound environment. In a corresponding wounded murine model with iron delivered intravenously, wound macrophages produced high levels of TNF and hydroxyl radical, and a senescence program was induced in nearby fibroblasts. As a result, wound closure was delayed.

The prolonged presence of the M1 phenotype is not the only macrophage-related problem that can contribute to wound healing disruption. In fact, if M2-like macrophages remain for too long, there may be excessive collagen formation, resulting in scarring (Sindrilaru and Scharffetter-Kochanek, 2013; Vannella and Wynn, 2017). It is interesting to note that fetal wound healing is scarless, with virtually no infiltrating macrophages, and many have attempted to mimic this model to improve wound healing outcomes in adults (Mackool et al., 1998). These examples suggest that an overabundance or prolonged presence of macrophages, regardless of the phenotype, can lead to wound healing complications.

## Experimental therapies and wound macrophages

Based on the role played by the different types of macrophages in the wound healing response, it has been hypothesized that interventions that dampen the M1 macrophage phenotype and promote M2-like characteristics may help the healing of chronic wounds. Some have even delivered exogenous macrophages as cell therapies for chronic wounds. A few of these approaches are highlighted below and summarized in Table 3. Note that the table focuses on key *in vivo* studies, whereas the text in the following subsections includes both *in vivo* and *in vitro* results.

**Table 3:** Experimental approaches to modulate macrophages in wound healing.

**Method**	**Wound model**	**Treatment details**	**Conclusion**	**References**
**ENDOGENOUS M1 ATTENUATION**				
Neutralizing Monoclonal antibodies: • anti-TNF-α • anti-F4/80 • control: non-specific, rat IgG	• ob/ob mice • Full-thickness excisional wounds (5 mm diameter)	• Systemic administration • 1 μg/g body weight • Day 7, 9 and 11 post-wounding (End of inflammatory phase)	• TNF-α and F4/80 antibodies effectively target and kill pro-inflammatory wound macrophages, resulting in accelerated healing	Goren et al., 2007
**EXOGENOUS M2 SUPPLEMENTATION**				
Injection of *in vitro* polarized: • M2a macrophages (by IL-4) • M2c macrophages (by-IL-10) • control: non-polarized macrophages (M0) • control: saline	• db/db mice • full-thickness excisional wounds (4 mm diameter)	• intradermal injection (0.5 × 10^6^ cells) • Day 1 and 3 post-wounding	•*In vitro*-polarized M2 macrophage supplementation immediately after wounding did not accelerate healing	Jetten et al., 2014
Ulcers treated with: • macrophages from blood of young, healthy donors, stimulated by hypo-osmotic shock (*n* = 72 ulcer patients) • conventional wound care (*n* = 127)	• human pressure ulcers in elderly patients • range of sizes (not indicated)	• intradermal injections near ulcer periphery and topically on ulcer • 0.05 mL/injection; 1 cm between injections along periphery • 2 × 10^6^ cells/mL • majority of ulcers treated 1 time; rare case of reinjection occurred 2 months after initial	• Injection of blood-derived macrophages to pressure ulcers resulted in healing of 27% of those treated vs. 6% in controls	Danon et al., 1997
Ulcers treated with: • macrophages from blood of young, healthy donors, stimulated by hypo-osmotic shock (*n* = 141 ulcers) • conventional wound care (*n* = 75)	• human pressure ulcers • human diabetic ulcers • large range in wound sizes; average ~30 cm^2^	• intradermal injections near ulcer periphery and topically on ulcer • 0.1 mL/injection; 1 cm between injections along periphery • 15–40 mL total depending on size • 2–4 × 10^6^ cells/mL • re-injection depending on wound condition ~4 weeks after initial treatment	• Injection of blood-derived macrophages lead to healing of a majority (69.5%) of pressure and diabetic ulcers compared to only 13.3% healed with standard treatment	Zuloff-Shani et al., 2010
**ENDOGENOUS MACROPHAGE MODULATION/M2 PROMOTION**				
**MSCs**				
Conditioned media from: • bone-marrow derived MSCS • control: fibroblasts	• healthy mice (Balb/C) • full-thickness excisional wounds (6 mm diameter)	• 100 μL total administered • subcutaneous (80 μL) and topical injections (20 μL)	• MSC-conditioned media resulted in increased numbers of macrophages and endothelial progenitor cells in the wound. Wound closure was significantly accelerated.	Chen et al., 2008
• human gingiva-derived MSCs (in PBS) • control: PBS	• healthy mice (C57BL/6J) • full-thickness excisional wounds (6 mm diameter)	• intravenous injection (2 × 10^6^ cells) • one time, on Day 1 after wounding	• Wound closure with MSC treatment was significantly accelerated. This occurred with a decrease in TNF-α and IL-6 and an increase in IL-10 and arginase-1	Zhang et al., 2010
Autologous bone-marrow derived: • MSCs • mononuclear cells • control: saline and standard care	• human diabetic ulcers • average size ~4 cm^2^	• intramuscular injection • 20 separate sites all on Day 1 • many cells used (exact number not clear)	• Ulcers treated with MSCs had accelerated healing compared to MNCs. Patients in this group also had better outcomes in terms of time to painless-walking, transcutaneous oxygen pressure and blood vessel formation	Lu et al., 2011
**Growth factors**				
• PDGF-BB • control: collagen-vehicle • control: non-irradiated	• healthy rats (Sprague-Dawley) • linear surgical incisions (6 cm long) • irradiated (whole-body or topically) to depress wound healing	• topical (2 μg and 10 μg/wound)• single dose	• Wounds treated with PDGF had higher cellularity scores and breaking strength. Effect of PDGF-BB was only seen in rats containing wound macrophages (topical irradiation vs. whole-body irradiation)	Mustoe et al., 1989
• recombinant human GM-CSF	• human chronic venous leg ulcers • range of sizes (not indicated)	• intradermal injection at 4 corners of wound • 150 μg	• GM-CSF causes wound macrophages to increase VEGF production, which results in improved vascularization in wounds	Cianfarani et al., 2006
**Biomaterials and macrophages**				
PEG-RGD hydrogels of varying stiffnesses: • 130 kPa • 240 kPa • 840 kPa	• healthy mice (C57BL/6)	• subcutaneous implantation • 5 mm diameter hydrogels	• Macrophage infiltration was the greatest in the stiffest hydrogels (840 kDa). Generally, stiffer hydrogels resulted in more severe foreign body responses.	Blakney et al., 2012
**HO-1 expression**				
Hemin (in diabetic rats) controls: • vehicle (in diabetic rats) • non-diabetic rats	• diabetic rats (streptozotocin-induced) • full-thickness excisional wounds	• topical 10% hemin ointment • daily	• HO-1 was induced in wounds of diabetic rats receiving hemin treatment. These wounds healed significantly faster than vehicle controls, at rates similar to non-diabetic rats. Hemin treatment led to decreased levels of TNF-α and IL-6 in wound tissue	Chen et al., 2016
• Hemin injection • topical povidone-iodine (positive control) • Saline injection	• healthy rats (Wistar) • full-thickness excisional wounds (2 × 2 cm^2^)	• hemin solution (diluted in saline) • intraperitoneal injection (30 mg/kg)• daily	• Hemin treatment increased wound closure and collagen synthesis. mRNA of pro-inflammatory cytokines ICAM-1 and TNF-α were decreased whereas anti-inflammatory IL-10 was increased. In some cases, the effect of hemin was greater than the positive control.	Ahanger et al., 2010
**Oxygen therapy**				
Hyperbaric Oxygen (HBO) Therapy • controls: normoxia hyperoxia increased pressure only	•*in vitro* human macrophage culture • cells stimulated with LPS, PHA, TNF-α, or Lipid A	• cells cultured in HBO, normoxia, hyperoxia, or increased pressure for up to 12 h	• Short-term (30 min) hyperbaric oxygen therapy (both increased pressure *and* oxygenation) has immunosuppressive effects on macrophages	Benson et al., 2003

### Endogenous M1 macrophage attenuation

Goren et al. (2007) aimed to silence M1 macrophages in obese/obese (ob/ob) mouse wounds (Goren et al., 2007). These animals have diabetes and hence, exhibit impaired wound healing. In the study, anti-TNF-α or anti-F4/80 antibodies were systemically administered beginning 7 days post-wounding, concurrent with the end of the inflammatory phase. These treatments resulted in wound closure and re-epithelialization while control wounds treated with a non-specific antibody remained unhealed with scabs. There were fewer total macrophages and decreased levels of TNF-α, IL-1β, and CCL2(MCP-1) proteins in wounds with anti-TNF-α and anti-F4/80. Furthermore, a greater proportion of wound macrophages were apoptotic compared to control groups. Overall, anti-TNFα and anti-F4/80 therapies reduced the impact of M1 macrophages, and accelerated the healing of diabetic wounds. It is noteworthy that by choosing Day 7 post-injury as the time point to begin treatment, necessary early inflammatory events in M1 macrophages were not disrupted. Treatment timing was strategically chosen to rescue the wound healing response during late-stage inflammation, during which the macrophage population should begin transitioning to M2.

### Exogenous M2 macrophage supplementation

Since the appearance of M2 macrophages correlates with a desirable progression in the wound healing response, direct addition of isolated M2 macrophages has been attempted to stimulate healing. However, as reported by Jetten et al. (2014), who used macrophages that were polarized into M2a and M2c phenotypes *in vitro* and then injected them into mouse wounds, this approach did not accelerate healing in wild type mice and even delayed healing in diabetic mice (Jetten et al., 2014). The M2 macrophages were introduced to the wounds during early inflammation (post-injury Days 1 and 3), and they continued to express M2 markers 15 days post-wounding. The lack of improvement in wound healing may be attributed to the timing of the treatment, which may have disrupted the function of M1 macrophages at a stage when they are presumably still needed. This study exemplifies the need to have an adequately-timed therapeutic approach.

In contrast, in Israel, treatment of chronic ulcers with blood-derived macrophages is an approved procedure, and it has been used successfully in over 1,000 patients (Zuloff-Shani et al., 2004). Danon et al. (1997), treated pressure ulcers in elderly patients with macrophages derived from blood units of young, healthy donors (Danon et al., 1997). The macrophage isolation method is completely sterile, using a closed system of interconnected bags containing the various reagents needed for the process. In order to stimulate isolated macrophages to produce factors beneficial for wound healing, they were activated by hypo-osmotic shock for 45 s (Zuloff-Shani et al., 2004). Related studies characterized these cells by measuring mRNA expression in over 72 genes (Frenkel et al., 2002). The results revealed that expression of several genes related to wound healing (IL-1, IL-6, TGF-β, FGF-8, TNF receptors, VEGF, and GM-CSF, to name a few) dramatically increased due to hypo-osmotic shock. Protein measurements revealed that hypo-osmotic shock could increase production up to 123- and 175-fold, in the case of IL-1 and IL-6, respectively, although donor-to-donor variability does exist. Hence, although this study did not utilize a traditional M2a/b/c/d-polarization method, macrophages were stimulated to be more anti-inflammatory via hypo-osmotic shock prior to wound application. However, the induced cell population was not completely characterized, particularly on the protein level.

In the clinical study, patients' ulcers were injected with the isolated macrophages near the wound periphery (Danon et al., 1997). A portion of the cell suspension was also deposited on top of the wound, which was then covered with dressings. Macrophage treatment was performed a single time in most patients, unless they still exhibited delayed healing about 1 month later, in which case a second treatment was performed. The effects of the treated ulcers were compared with other patient ulcers at the same hospital treated with conventional methods, including debridement, antibiotics and wound dressings. Results revealed that 27% of ulcers treated with macrophages healed, whereas only 6% of controls did, and that there were no adverse reactions to treatment.

The same group later published results of a more comprehensive study, including randomization of patients between macrophage-treated and standard-of-care groups (Zuloff-Shani et al., 2010). In addition to providing more data, including healing time, etiology, and size of the wounds in this study, subsets of patients with diabetic ulcers were also included and analyzed separately. The overall results for all ulcers demonstrated improved statistics compared to the previous study: 69% of macrophage-treated patients healed in an average of 86.7 days, whereas control groups had only 13.3% full-closure wounds in 117.7 days. Similarly, in the diabetes groups, 65.5% of wounds with the macrophage treatment and only 15.4% of controls healed. Again, wounds in the treatment group healed in a faster time compared to controls.

These studies provided an interesting strategy of using exogenous macrophages from healthy individuals, stimulated by hypo-osmotic shock, without the use of LPS, IFN-γ, or any other stimulus, to aid in the healing of chronic ulcers. The success of the treatments in both pressure and diabetic ulcer patients is promising, however more work must be done to determine the reason why some wounds do not respond to treatment, and investigate ways to improve these outcomes. Additionally, isolation and purification of macrophage populations was not extensive in these studies, and therefore cell types other than monocytes/macrophages may be contributing to this therapy.

### Endogenous macrophage modulation/M2 phenotype promotion

Several different approaches have been taken to modulate wound macrophages with the goal of promoting M2-like characteristics, which often simultaneously attenuate M1 characteristics. Although not a comprehensive list, methods using mesenchymal stromal cells (MSCs), growth factors, biomaterials, heme oxygenase-1 (HO-1) induction, and oxygen therapy are discussed.

#### Mesenchymal stromal cells

Mesenchymal stromal cells (MSCs) secrete many growth factors that are required for wound healing, and have therefore been explored as cell therapies. Their use in animal and human studies has been successful, resulting in accelerated wound closure and more mature angiogenesis and granulation tissue (Nuschke, 2014). Evidence shows that MSCs and their secreted products affect a variety of skin and immune cells. Of particular interest to this review are MSC interactions with macrophages. MSCs have such powerful modulating effects on macrophages, that some have defined yet another phenotype of macrophages based on this interaction (Kim and Hematti, 2009). These MSC-educated macrophages exhibit M2-like characteristics (IL-10 high, IL-12 low, IL-6 high, TNF-α low) and hence possess a secretome that can have powerful benefits in wound healing.

One of the mechanisms of MSC action on wounds is via recruitment of macrophages. Chen et al. (2008) used MSC-conditioned media *in vitro* and found that it accelerated migration of macrophages, in addition to keratinocytes and endothelial cells (Chen et al., 2008). In a murine excisional wound model, subcutaneous injection and topical application of the MSC-conditioned media also led to increased presence of macrophages and endothelial progenitor cells. Macrophage recruitment by MSCs may be attributed to high levels of secreted chemoattractants CCL3(MIP-1α), MIP-2, and CCL12(MCP-5).

MSCs also secrete an important regulator, prostaglandin E-2 (PGE-2), that has a direct effect on macrophages, by reprogramming them to up-regulate the M2-like marker, IL-10 (Nemeth et al., 2009; Barminko et al., 2014). In a murine sepsis model, Nemeth et al. (2009) showed that systemic MSC administration reduced mortality and improved organ function, but only in the presence of macrophages (Nemeth et al., 2009). When macrophages were depleted, the benefits of the MSC-treatment were eliminated. In response to the MSC-treatment, extracted lung macrophages produced significantly higher IL-10 (an M2-like marker) compared to those from control groups. As a result, neutrophil tissue infiltration was decreased, which has a protective effect due to lower levels of local oxidative tissue damage. The group also performed *in vitro* studies to determine the molecular interaction between MSCs and macrophages that leads to IL-10 upregulation. Results suggested that PGE-2 from MSCs stimulates macrophages to produce IL-10. Similar findings were confirmed *in vitro* by Barminko et al. (2014), showing that MSCs, via PGE-2, reduced TNF-α and increased IL-10 secretion from macrophages, hence attenuating M1, and promoting M2, characteristics (Barminko et al., 2014). Although these studies were not performed in a chronic wound model, they reveal relevant interactions between MSCs and macrophages, which may partially explain the success of MSC therapy in wound healing studies.

In a wound healing context, MSCs have a similar effect on macrophages. Zhang et al. (2010) studied the effect of human gingival-derived MSCs on macrophage phenotype and found that *in vitro*, they increased expression of M2-like markers IL-10, IL-6, and CD206, decreased expression of TNF-α (M1-like marker) and decreased induction of Th-17 cell expansion (Zhang et al., 2010). In an *in vivo* murine wound healing model, systemically-administered MSCs accumulated near the wound area, and induced M2 characteristics in surrounding macrophages, such as increased IL-10 and decreased TNF-α and IL-6. Wound healing was accelerated with MSC-treatment.

Several clinical studies have also investigated the potential of MSCs as a treatment for chronic wounds. As discussed in the previous section, exogenous monocyte/macrophage cell therapies have also been tested in human wounds (Danon et al., 1997; Zuloff-Shani et al., 2010). One interesting clinical study compared the effects of MSCs vs. mononuclear cells (MNCs) in diabetic ulcers (Lu et al., 2011). Both cell sources were autologous and bone-marrow derived. MSCs were expanded *in vitro*, whereas the mononuclear fraction—containing a variety of hematopoietic cell types including monocytes— was isolated from bone marrow aspirate. Prior to administration to ulcers, MSCs and MNCs were analyzed for levels of angiogenic factors. Interestingly, MSCs produced significantly higher levels of VEGF, FGF-2, and angiopoeitin-1 than MNCs in both hypoxic and normoxic conditions. In the clinical study, ulcers treated with MSCs healed significantly faster, and were fully closed 4 weeks earlier than treatment with MNCs. The MSC-group also had the best outcomes in terms of pain-free walking time, transcutaneous oxygen pressure, and blood vessel formation, followed by the MNC-group and, lastly, the saline controls. These results showed that MSCs had more potent effects on diabetic wounds compared to MNCs. This suggests that MSCs, rather than a monocyte-based treatment, may have more potent effects in a wound environment. Another possibility is that the MNC group may have performed better if it was stimulated, for example by hypo-osmotic shock, or pre-polarized into an M2-like phenotype via biochemical stimulation. Regardless, MSCs are known to have powerful modulating effects on macrophages, therefore this approach may be better-suited for wound healing compared to monocyte/macrophage supplementation, as suggested by these results.

In developing new therapies, it is pertinent to consider the special characteristics of chronic wound environments, such as low oxygen tension, and how they may affect macrophage function. Through *in vitro* studies, Faulknor et al. (2017) demonstrated that a hypoxic environment lessened macrophage plasticity in response to MSCs (Faulknor et al., 2017). Macrophages cultured in normoxic conditions (20% O_2_) with MSCs produced high levels of the M2 marker, IL-10, however, in hypoxic conditions (1% O_2_), secretion was significantly lower. As macrophages possess a high degree of phenotypic plasticity, they react not only to the treatments that are introduced, but also to the existing microenvironment, which may affect their ability to respond to treatment. This is an important consideration that emerging chronic wound therapies should address.

#### Growth factors

Cell therapies provide wounds with numerous cytokines and growth factors, which in sum affect local cells and enhance the coordinated wound healing process. Another approach to treating chronic wounds is through the application of a single growth factor, which can elicit cellular responses. As advertised on their website, Regranex is the, “first and only FDA-approved recombinant PDGF therapy for diabetic neuropathic ulcers” (Smith, 2018). The mechanism of action involves macrophages as a key player. During hemostasis, PDGF recruits macrophages to the wound in order to initiate inflammation. In the next phase, macrophages are stimulated by Regranex to produce more PDGF, as well as TGF-β, to stimulate extracellular tissue formation by fibroblasts. An early study on surgical incisions in rats investigated the mechanism of PDGF therapy by studying its effects in rats receiving either surface irradiation or total body irradiation (Mustoe et al., 1989). Whereas, surface-irradiated rats retain bone marrow elements and wound monocytes/macrophages, total body irradiated rats are depleted of them. Hence, this approach was used to determine the importance of macrophages in the efficacy of PDGF in wound healing. The results revealed that PDGF therapy was ineffective in aiding wound healing in rats depleted of monocytes/macrophages (total body irradiation). In contrast, in rats that were surface-irradiated, macrophages were able to migrate into the wounds and PDGF treatment successfully aided healing. The number of wound fibroblasts increased, as well as wound strength, presumably by the formation of more collagen. Interestingly, in wounds that contained fibroblasts but no macrophages (total body-irradiated rats), PDGF did not stimulate collagen synthesis. This suggests that macrophages are the first-responders to PDGF treatment, and in response, they must activate fibroblasts, via TGF-β, to proliferate and synthesize collagen, which contributes to granulation tissue formation and wound closure. Overall, wound macrophages are a vital part in the mechanism of action of Regranex.

Interestingly, granulocyte macrophage-colony stimulating factor (GM-CSF) has also shown benefits in chronic wound healing (Da Costa et al., 1999), despite the fact that it is used *in vitro* to promote the M1 phenotype. In cell culture, GM-CSF induces macrophages to produce more pro-inflammatory factors (TNF, IL-23) and less anti-inflammatory factors (IL-10) compared to baseline levels, however, LPS and IFN-γ are often used as more potent M1-stimuli that activate different signaling pathways (Lacey et al., 2012). In contrast, *in vivo* effects of GM-CSF, particularly in a chronic wound healing environment, can promote healing. Cianfarani et al. (2006) demonstrated that GM-CSF injections to non-healing venous leg ulcers induced VEGF transcription in the wound bed, primarily within macrophages (Cianfarani et al., 2006). As PDGF, in the previous example, stimulates macrophages to produce TGF-β, GM-CSF stimulates macrophages to produce VEGF. This finding was corroborated with *in vitro* results showing the same effect of GM-CSF on VEGF production in a differentiated monocytic cell line but not in keratinocytes. In patient ulcers, increased VEGF transcription by GM-CSF lead to improved vascularization and healing. GM-CSF also acts on other skin cells, which further explains its pro-wound healing effects. In addition to macrophages, GM-CSF also has chemotactic effects on fibroblasts, endothelial cells and keratinocytes. Accordingly, formation of granulation tissue, blood vessels and the epidermal layer are improved with exogenous GM-CSF (Mann et al., 2001).

A potential explanation of the pro-wound healing effect of GM-CSF *in vivo* vs. its perceived pro-inflammatory role *in vitro* is that, within the complex chronic wound environment, which contains several interacting cell types and signaling pathways, an intermediate macrophage phenotype is formed. A combination of M1-like and M2-like factors, such as IFN-γ, IL-6 and TGF-β are increased upon upregulation of GM-CSF *in vivo*, all of which have distinct roles in the wound healing process (Mann et al., 2001). Interestingly, evidence shows that GM-CSF is more effective in accelerating chronic wound healing rather than acute (Barrientos et al., 2014). This discrepancy further underlines the complex role of macrophages within the intricate, multi-cellular wound healing environment.

Many other growth factors (i.e., VEGF, FGF, EGF) have shown potential in aiding in wound healing (Barrientos et al., 2014), however less work has been published showing their direct effect on macrophages, as they primarily act on other cells such as endothelial cells, fibroblasts, and keratinocytes. Regardless, FGF and EGF are approved wound care therapies in Japan and Cuba, respectively (Frykberg and Banks, 2015).

#### Immunomodulatory biomaterials

Any material that comes in contact with the body has the potential to elicit an immune response and affect surrounding cells and tissue. The body's response to the material is not always harmful, and can be tuned to promote healing if the material possesses the right characteristics. Immunomodulatory materials are being developed with the goal of limiting negative reactions to implants and instead, promoting their integration into the body (Vishwakarma et al., 2016). General approaches when designing immunomodulatory materials include (1) carefully selecting physical properties, (2) altering chemistry, (3) incorporating therapeutic molecules for controlled release and, (4) combination with cell therapies. As the last two points were discussed in previous sections, the following discussion is focused on physical and chemical properties of biomaterials that modulate macrophage behavior. The discussion includes examples of both currently used wound healing materials, and those under development for potential future applications.

One chemical approach involves modifying native extracellular matrix molecules. Hyaluronan (HA), a glycosaminoglycan (GAG), is one such ECM component that can cause macrophages to take on pro- or anti-inflammatory characteristics depending on certain chemical modifications (Stern and Maibach, 2008; Vishwakarma et al., 2016). For example, sulfated GAGs can bind and interact with growth factors and cytokines, thereby preventing them from affecting macrophage behavior. Kajahn et al. (2012) tested the *in vitro* response of monocytes to biomaterials composed of collagen and HA, or sulfated HA derivatives (made by simultaneously degrading and sulfating native HA), within an inflammatory environment created by exogenous IL-6, IFN-γ, and MCP-1 (Kajahn et al., 2012). In the presence of collagen with highly-sulfated HA derivatives, monocytes resisted an M1 phenotype transition [via lower levels of IL-1β, CXCL8(IL-8), IL-12 and TNF-α], and instead differentiated into M2-like macrophages with increased IL-10 production and CD163 expression. Other experimental conditions, including collagen only, collagen + non-sulfated HA, and collagen + lowly-sulfated HA derivatives, promoted macrophages with more M1 characteristics. The results of this study are interesting in regards to wound healing, as several wound-care products are based on ECM proteins.

Chitosan is another material that is in found in several FDA-approved wound products (Dai et al., 2011). It is known for its antimicrobial effects and also acts on skin cells to aid in wound healing. Researchers have also investigated its effect on macrophages (Ueno et al., 2001). In response to culturing with chitosan, macrophages increased production of TGF-β1, which stimulates ECM formation. In contrast, chitosan did not stimulate direct ECM formation by fibroblasts. This result highlights the importance of macrophage subset modulation as they produce many growth factors that can affect other local wound cells. Additionally, chitosan also stimulated macrophages to produce high levels of PDGF, which is important in angiogenesis. Other studies have shown that chitosan promotes nitric oxide production and chemotaxis in macrophages (Peluso et al., 1994). It is believed that the cellular interaction occurs via N-acetylglucosamine on chitosan and corresponding receptors on macrophages.

Physical cues on biomaterials can also affect macrophages by causing them to take on rounded vs. elongated shapes, which are likely to exhibit M1- or M2-like characteristics, respectively (McWhorter et al., 2013). One approach to achieving M2-like macrophages on biomaterials is by micropatterning ECM molecules or integrins that promote cell attachment and spreading (McWhorter et al., 2013; Cha et al., 2017). Modifications like these can also alter the stiffness of the cell/biomaterial interface. Blakney et al. (2012) investigated the effect of hydrogel stiffness and macrophage adhesion in an *in vivo*, subcutaneous implantation murine study (Blakney et al., 2012). All hydrogels (130, 240, and 840 kPa moduli) were composed of polyethylene glycol and RGD, to allow for cell attachment. Hydrogels were implanted into mice, and 28 days later, were removed for histological analysis. Staining with a macrophage-specific cell-surface marker, Mac3 (CD107b), revealed that the softest hydrogels had significantly lower macrophage infiltration compared to the other two groups. These results suggest that stiffness of wound care products may be important in directing macrophage fate, and overall wound healing success.

In some cases, a combination of chemical and physical cues and an understanding of which has the greater effect, can further promote differentiation of the desired macrophage phenotype (McWhorter et al., 2013; Cha et al., 2017). This can be optimized by intentional selection of material properties to achieve successful immunomodulatory biomaterials. Surprisingly, many studies in the literature seem to consider immunomodulatory properties of wound dressings and therapies as an afterthought with their experimental treatment or product. Recognizing the importance of macrophages in the wound healing process, it is pertinent to ensure that a material that is introduced to chronic wounds does not further promote a pro-inflammatory environment, but rather attenuates M1 macrophages and promotes the transition to M2-like phenotypes. Moving forward, immunomodulatory properties of materials should be a key design factor for new wound healing therapies.

#### Heme oxygenase-1 induction

Heme oxygenase-1 (HO-1) is an inducible enzyme that catalyzes heme breakdown and releases anti-inflammatory factors. When hemoglobin is endocytosed by macrophages, the HO-1 pathway breaks it down into iron, carbon monoxide and biliverdin, which is converted to bilirubin.

The three products of HO-1 activity can individually affect wound healing responses. Carbon monoxide and bilirubin can exert anti-inflammatory properties to help wound healing (Kapitulnik, 2004; Ryter et al., 2006) however, differential regulation of the iron product can promote M1- or M2-like macrophages (Cairo et al., 2011). M1-like cells store the majority of the iron intracellularly as ferritin, whereas, M2-like macrophages release it to the extracellular environment via the transmembrane channel, ferroportin. Likewise, M2-like macrophages express higher levels of ferroportin compared to M1. M2-like cells have a higher number of hemoglobin-binding receptors (specifically CD163). Thus, the HO-1 signaling pathway is more active. Perhaps the downstream effects of this process, such as higher HO-1 activity, carbon monoxide and bilirubin production, and iron release in M2 macrophages contributes to their pro-regenerative properties.

The HO-1 pathway is important in wound healing, as it plays roles in angiogenesis and re-epithelialization (Grochot-Przeczek et al., 2009). Mice with inhibited or deleted HO-1 exhibit delayed wound healing, and diabetic mice inherently have lower levels of HO-1, which may partially explain their challenges with wound healing. Restoring HO-1 expression in wild type and diabetic mice resulted in improved and accelerated wound healing, which suggests an important role of hemoglobin breakdown in the wound healing process. HO-1 is also expressed in fibroblasts and keratinocytes, which underlines its role in dermal wound healing (Lundvig et al., 2012).

During acute wound healing, HO-1 protein was expressed at high levels in a murine model 3 days after creation of full-thickness excisional wounds, before returning to basal levels (Hanselmann et al., 2001). HO-1 mRNA levels continued to be high until Day 7. Macrophages and proliferating keratinocytes along the wound edge were the primary cell types that overexpressed HO-1. Interestingly, it was also found that patients with psoriatic skin constitutively overexpress HO-1, as well as HO-2. *In vitro* studies found that ROS, rather than growth factors or cytokines (KGF, EGF, TNF-α), directly stimulated HO-1 expression.

HO-1 has also been targeted in models of delayed wound healing. In a wounded diabetic rat model, HO-1 expression was induced using topical, 10% hemin ointment (Chen et al., 2016). Wound TNF-α and IL-6 levels, as measured by Western blot, were significantly decreased compared to rats treated with vehicle controls. VEGF and intercellular adhesion molecule (ICAM) serum levels were increased, and accordingly, so was blood vessel density. Induction of HO-1 in diabetic rats brought levels of several measured biomolecules, as well as wound healing rates, back to those seen in non-diabetic controls. Even in non-diabetic rats, studies have shown that hemin accelerates healing, concurrently with decreased levels of pro-inflammatory proteins ICAM-1 and TNF-α and increased IL-10 (Ahanger et al., 2010). The involvement of these prototypical M1/M2 markers suggests the involvement of macrophages in the enhancement of wound healing.

In fact, there is evidence that HO-1 expression in macrophages promotes an M2-like phenotype (Mhem) (Naito et al., 2014). Several animal studies in different disease models, have induced HO-1 expression and measured subsequent macrophage markers. Resulting M2-like markers include arginase-1, mannose receptor, and CD163, among others. HO-1 has demonstrated potential as a method to promote M2-like characteristics in macrophages to aid in healing, however, as with many macrophage-targeted therapies, timing must be well-suited in order to successfully resolve inflammation (Lundvig et al., 2012).

Hemoglobin-based substances (polymerized hemoglobin, PEG-encapsulated hemoglobin, etc.) may be interesting approaches to activate the HO-1 pathway, while simultaneously delivering oxygen (Palmer et al., 2009; Palmer and Intaglietta, 2014). This method would elicit anti-inflammatory effects from local macrophages, and restore oxygen levels, thereby targeting two major deficiencies of chronic wounds with one therapy.

#### Oxygen therapy

Chronic wounds are hypoxic, as blood flow and, hence, oxygen delivery to the tissues are disrupted (Sen, 2009). Direct delivery of oxygen to skin wounds, such as by exposing patients to 100% oxygen at 2–3 atm pressure in hyperbaric chambers, has been shown to enhance wound healing. The effect of increasing oxygen levels in the wound are multifaceted, but evidence suggests that one of the targets may be the wound macrophages. One study investigated the direct effects of hyperbaric oxygen and hyperoxia (without increased pressure) on the cytokine profiles of cultured macrophages (Benson et al., 2003). Hyperbaric oxygen dampened IL-1β and TNF-α secretion by ~40%, while hyperoxia alone had no effect. However, when hyperbaric oxygen exposure exceeded 6 h, an increase, rather than a decrease, in the production of these pro-inflammatory mediators was observed. This study did not investigate any M2-like macrophage functional parameters.

## Conclusions and future directions

It is clear that macrophages play an important role in wound healing, and that anti-inflammatory, M2-like phenotypes are desirable for efficient healing. Questions remain regarding the details behind monocyte recruitment and macrophage differentiation, specifically whether monocytes are predestined to become one particular phenotype (M1/M2-like) or if macrophages themselves change from M1 to M2 phenotypes (or vice versa) within the tissue (Figure 1; Vannella and Wynn, 2017). More thorough histological studies on *in vivo* wound environments (both acute and chronic) would lead to a better understanding of macrophage phenotypes and their spatiotemporal and functional contributions during healing. This information could help identify macrophage phenotypes needed to promote healing in chronic wounds. Another challenge in this field is that the definition of each macrophage sub-phenotype is neither clear, nor agreed upon. There are also inconsistencies between *in vitro* and *in vivo* macrophage phenotypes, especially in chronic wound models, which further confuse this area of research. There is a need for a more thorough characterization of macrophage phenotypes and a definition of their respective roles (Table 2 and Figure 2). Novel technologies and tools that can quickly and thoroughly define macrophage phenotypes, even within heterogeneous populations, would advance research (Ginhoux et al., 2016; Murray, 2017). In the midst of this work, it is also important to recognize differences between murine and human wound healing processes and roles of immune cells (Pasparakis et al., 2014).

Current experimental therapies that are being investigated for their potential to promote wound healing macrophages include mesenchymal stem cells, growth factors, biomaterials, and more (Table 3). Up-and-coming methods to control macrophage fate include microRNA therapies to affect macrophage transcriptome and function (Self-Fordham et al., 2017). Delivery time for novel therapies, in regards to current macrophage phenotype and the needs of the particular wound, should not be overlooked, as it can make the difference between an effective and an ineffective therapy. Another question is whether or not directly promoting M2-like phenotypes is entirely necessary, or, is it possible that by only attenuating M1 macrophages, the wound environment will be reprogrammed to successfully heal? Furthermore, is targeting macrophages alone enough to promote healing, within the complex, multi-cellular chronic wound environment? Hence, an effective treatment may need to address multiple deficiencies of chronic wounds. As macrophages are involved in all phases of wound healing, and their dysregulation in chronic wounds leads to a stalled and heightened inflammatory state, an improved understanding of these key regulators will ultimately lead to advancements in wound healing therapies.

## Author contributions

PK wrote the majority of the review text and generated the figures. AP, RS, and FB read and edited several drafts, provided valuable feedback and suggested ideas and broad perspectives to address in the review.

### Conflict of interest statement

The authors declare that the research was conducted in the absence of any commercial or financial relationships that could be construed as a potential conflict of interest.

## Abbreviations

DAMPs: damage/danger-associated molecular patterns
ECM: extracellular matrix
EGF: epidermal growth factor
FGF: fibroblast growth factor
GC: gluococorticoid
GM-CSF: granulocyte-macrophage colony-stimulating factor
GT: granulation tissue
HA (in text): hyaluronan
HA (in table): hemorrhage-associated
HO-1: heme oxygenase-1
IFN-γ: interferon-gamma
IGF-1: insulin-like growth factor 1
IL-(1β, 6, 10, etc.): interleukins
iNOS: inducible nitric oxide synthase
LPS: lipopolysaccharide
M-CSF: macrophage colony-stimulating factor
MCP-1/5: monocyte chemoattractant protein-1/5 (CCL2/CCL12)
MHCII: major histocompatibility complex class II
MIP-1α/β: macrophage inflammatory protein-1 alpha/beta (CCL3/CCL4)
MNCs: mononuclear cells
MMPs: matrix metalloproteinases
MSCs: mesenchymal stromal cells
NF-κβ: nuclear factor kappa beta
PAMPs: pathogen-associated molecular patterns
PDGF: platelet-derived growth factor
PGE-2: prostaglandin E-2
ROS: reactive oxygen species
TAMs: tumor-associated macrophages
TGF-β1: transforming growth factor beta 1
TNF-α: tumor necrosis factor-alpha
VEGF: vascular endothelial growth factor.
